# The Approaching General Election

**Published:** 1905-12-16

**Authors:** 


### The Hospital.

A Journal of

The Medical Sciences and Hospital Administration.

Vol. XXXIX.—No. 1,003. SATURDAY, DECEMBER 16, 1905.

### The Approaching General Election.

The resignation of Mr. Balfour and the appointment of Sir Henry Campbell-Bannerman as his successor must of necessity bring about an immediate dissolution of Parliament, inasmuch as it would be manifestly useless for the new Prime Minister to meet a House of Commons in which his opponents still command a considerable majority. It follows that, some time during January at the latest, the country will be plunged into the turmoil of a General Election, and that we shall soon be inundated with the manifestoes of opposing candidates. In the present state of political parties, and in view of the obvious determination of the leading Irish politicians to cast their weight into whatever scale may appear most favourable to their attainment of some measure of Home Rule, the programme of the incoming ministers and of their followers among parliamentary candidates will not be very easy of construction; and it will be interesting to observe in what proportion of cases any attempt will be made to balance ambiguities in other directions by clear pronouncements of opinion on the great sanitary questions which are of real national importance, and compared with which the trumpery quarrels of the ordinary "ins" and "outs" are scarcely of more weight than the sports of children. It is, of course, of serious, and it may even be of paramount importance that the foreign relations of the country should be committed to the hands of a statesman who will be prepared to maintain, in all essentials, the policy which has been so successful during the last ten years, and who belongs to the class with which the Ministers of foreign countries are accustomed not only to transact business but also to establish personal relations of a friendly and confidential character. Admitting this to the full, and resting upon what has now become a reasonable hope that the desired conditions will be fulfilled, the remaining questions before the country will be very largely those which have a direct bearing upon public health; and it will be exceedingly desirable that the electors should seek for clear pronouncements concerning them from all who ask for support at the polls. Stating them in the order of their importance, these questions appear to be the reform and better administration of the Poor Law, the improvement of education, the promotion of temperance, and the exercise of sufficient pressure to compel county councils, municipalities, and other popularly elected bodies, to carry the provisions of the various Public Health Acts into effect. It will probably be admitted by all who are conversant with the facts that the machinery of local government in relation to health has very largely broken down; and that municipal and other electors have not risen to any adequate conception of the magnitude of the trust reposed in them. Their ignorance or indifference has been the direct cause of great disasters, such, for example, as the epidemics of typhoid fever at Maidstone, Worthing, Lincoln, and Basingstoke. The chief duties of government are to protect the persons and the property of the governed; and it is no answer to a charge of neglecting to protect persons that power to do this was given to somebody else, say to a municipal corporation, and that the members of that corporation likewise failed to discharge the duties committed to them. It is the duty of the Government to protect the life of the humblest member of the minority of electors, say at Lincoln or at Basingstoke; and the Government is not released from that duty by the fact that the majority chose to elect incompetent or negligent persons to be town councillors. Instead of its being released, the duty of protecting the minority may often be said to have become more binding; for, in the great majority of instances, it will be found that the incompetent persons have been elected to municipal office mainly upon the ground of their real or supposed adhesion to some political party, and upon their skill in pronouncing the phrases which constitute its shibboleth. Apart from this, their sole claim would too often rest upon the fact that they were themselves the chief proprietors of the local nuisances, and that they felt a deceptive and short-sighted interest in maintaining them in complete activity. Security of tenure to Medical Officers of Health, and power to them to carry the defaults of municipal authorities to a higher court, would be certain, in the course of a very short time, to bring about a great reduction in the mortality of the kingdom.

The administration of the Poor Law must be expected to remain very much in its present condition until time has been afforded for the recently appointed Royal Commission to report, and for legislation to be founded upon the disclosures which its report may be expected to contain. It would be very difficult, in the meanwhile, for individuals to obtain a sufficiently comprehensive view of the vast field of the inquiry; but it may be asserted with some confidence that the facts have outgrown the legislation of seventy years ago, and that the experience gained during the interval should be sufficient, if properly garnered and employed, to throw much light upon the principal conditions of the problems relating to poverty with which the nation is now confronted, and which threaten to become larger and more complicated as time goes on. Even if we put aside the economical aspects of the question, and regard it only from the comparatively narrow point of view of public health, it is manifestly of vast and far-reaching importance. We have an uncertain number of persons, of both sexes, in whom lack of employment has sapped the habit of productive industry, and who, through their progeny even more than in themselves, constitute a constantly increasing source of national weakness and degeneracy, physical as well as moral. They swell the ranks of the diseased and of the intemperate, of idleness, of immorality, and of crime. Their presence in any locality is generally in direct proportion to the laxity of its relief administration, although this may be due to migration as well as to direct production. They shut out the genuine unemployed from popular sight and sympathy, and they constitute a nuisance with which it is imperatively necessary for the legislature to deal. It may be doubted whether the new Ministry will retain office long enough for the purpose; but, however that may be, it is high time that public attention should be directed to the requirements of the case, and that a demand should arise for their fulfilment.

